# Assessment of the scope and practice of evaluation among medical donation programs

**DOI:** 10.1186/s12992-016-0210-8

**Published:** 2016-11-04

**Authors:** Alisa M. Jenny, Meng Li, Elizabeth Ashbourne, Myron Aldrink, Christine Funk, Andy Stergachis

**Affiliations:** 1 Department of Global Health, Global Medicines Program, University of Washington, Harris Hydraulics Building, Room 321, 1705 NE Pacific St., Box 357965, Seattle, WA 98195-7965 USA; 2 Department of Pharmacy, Pharmaceutical Outcomes Research and Policy Program, University of Washington, Seattle, WA USA; 3 Partnership for Quality Medical Donations, Annapolis, MD USA; 4 Pacific Links LLC, Grand Rapids, MI USA; 5 Merck, Kenilworth, NJ USA

**Keywords:** Medical Donations, Low- and Middle-Income Countries, Neglected Tropical Diseases, Monitoring, Evaluation, Impact

## Abstract

**Background:**

Medical donation programs for drugs, other medical products, training and other supportive services can improve access to essential medicines in low- and middle-income countries (LMICs) and provide emergency and disaster relief. The scope and extent to which medical donation programs evaluate their impact on recipients and health systems is not well documented.

**Methods:**

We conducted a survey of the member organizations of the Partnership for Quality Medical Donations (PQMD), a global alliance of non-profit and corporate organizations, to identify evaluations conducted in conjunction with donation programs.

**Results:**

Twenty-five out of the 36 PQMD organizations that were members at the time of the survey participated in the study, for a response rate of 69 %. PQMD members provided information on 34 of their major medical donation programs. Half of the donation programs reported conducting trainings as a part of their donation program. Twenty-six (76 %) programs reported that they conduct routine monitoring of their donation programs. Less than 30 % of donation programs were evaluated for their impact on health. Lack of technical staff and lack of funding were reported as key barriers to conducting impact evaluations.

**Conclusions:**

Member organizations of PQMD provide a broad range of medical donations, targeting a wide range of public health issues and events. While some level of monitoring and evaluation was conducted in nearly 80 % of the donation programs, a program’s impact was infrequently evaluated. Opportunities exist to develop consistent metrics for medical donation programs, develop a common framework for impact evaluations, and advocate for data collection and analysis plans that collect meaningful metrics.

## Background

Global access to quality medicines and other medical products is fundamental to maintaining and improving the health of people. Maintaining a reliable supply chain of essential medicines and other medical products can save lives, reduce morbidity, and improve quality of life. Unfortunately, poor availability of medicines and other medical products in many low- and middle-income countries (LMICs) where health systems, including supply chains, are commonly suboptimal. Numerous studies have described a lack of availability of essential medicines in LMICs [1–5]. Moreover, poor quality medicines are a global health problem, particularly in LMICs, resulting in the potential for treatment failure, development of antimicrobial resistance, and serious adverse drug reactions, increasing healthcare costs and undermining the public’s confidence in healthcare systems [6, 7].

The situation of poor access to medicines and other medical products in LMICs is further compounded when those countries are struck by natural disasters, such as typhoons, hurricanes, tsunamis or earthquakes, which put an even greater strain on their weak health systems. Among the top 10 countries in terms of disaster mortality in 2014, seven countries are classified as low income or lower-middle income countries [8]. In response to these needs and concerns, nongovernmental organizations (NGOs) and pharmaceutical and medical supply manufacturers are involved in performing various aspects of donations, including delivery and/or distribution of medical products and devices, and in-country training and coordination activities [9, 10].

Donations of medicines and other medical products are a key component of medical relief efforts, and represent a global response to countries and regions affected by human and natural disasters [11]. Previously, there was a widespread belief that any medicine is better than none. However, reports of many unannounced, inappropriate, and unused donations to Bosnia-Herzegovina and Croatia during war lead the World Health Organization (WHO) to issue guidelines for international drug donations in 1996 [12–16]. The WHO guidelines were subsequently updated in 1999 and 2010, including adding a section on monitoring and evaluation of drug donation programs that focuses on evaluating the appropriateness of medicine donations [12]. This section notes the importance of “assessments of the administrative process used by the donor agency, the adequacy of selection and forecasting, appropriateness of the medicines, timeliness of delivery and changes in treatment guidelines.” The guidelines also recommended using cost-benefit analysis to help determine the donation’s “usefulness” to the donor and the recipient.

However, a search of peer-reviewed literature yielded few studies that evaluated the impact of medical donations. In a review of Medline (1946-May 2015) and EMBASE (1996-May 2015), the authors found only five impact evaluations of specific donations, all focusing on large donation programs [17–21]. One of the best known examples is the Mectizan® Donation Program by Merck for treatment of river blindness and lymphatic filariasis [22, 23]. There are also a few reports of the effects of drug donation programs in the form of monographs [24, 25]. Additionally, there are a few published economic evaluations of medical donation programs [26–29]. Given the size and scale of global donation programs, there is a need for more impact assessments and greater consistency and transparency in reporting performance metrics.

The Partnership for Quality Medical Donations (PQMD) is a global alliance of non-governmental organizations and leading pharmaceutical companies, seeking to enhance access to healthcare in underserved communities and areas affected by disaster. Data collected from PQMD in 2015–16 estimated that over $3 billion in medical donations were provided as part of regular donation programs, as well as donations in response to the earthquake in Nepal, and the outbreaks of Ebola, and Zika [30]. PQMD has published guidelines for medical donation programs, which include the need for monitoring and evaluation of donations to measure the effects, both long- and short-term, and to learn from successes and challenges [31]. Given the lack of published data on the impact of global medical donations, the goal of our study was to understand the scope of medical donation programs and assess how monitoring and evaluation and impact evaluations have been carried out among PQMD members.

## Methods

We conducted a survey of PQMD member organizations to better understand the scope of their donation programs and the types of data currently being collected as part of routine monitoring and evaluation activities, as well as any impact evaluations that have been conducted as part of a medical donation program. The survey asked respondents to describe up to three major donation programs offered by their organizations. Questions about donation programs included when each program was initiated, types of events targeted by the donation, types of products donated and the geographical regions served. Respondents were then asked to describe prior and ongoing monitoring and evaluation activities, including any impact evaluations conducted by the organizations.

**Fig. 1:**
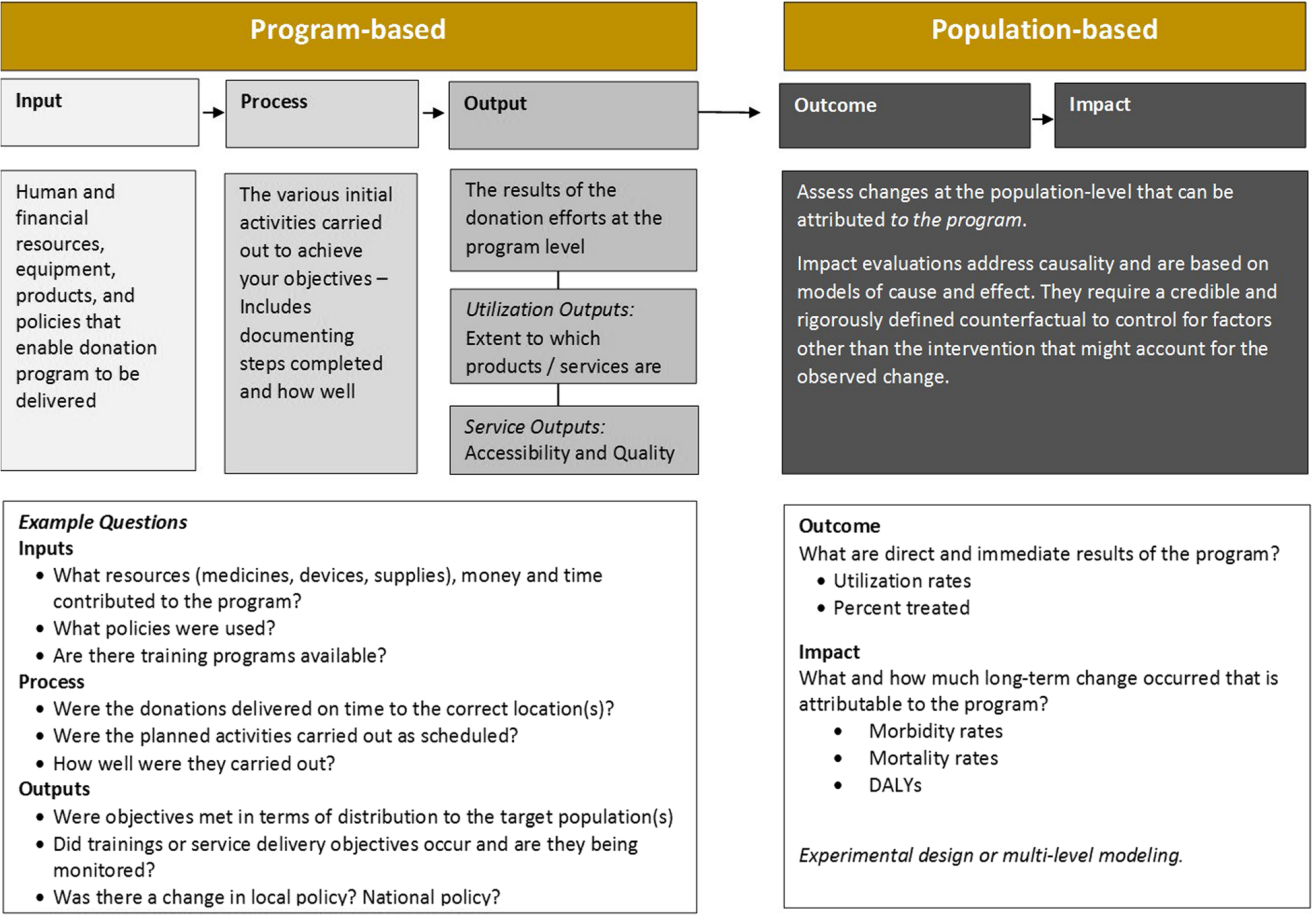
Framework for Measuring Impact of Medical Donations

To guide the design of the survey, we developed a conceptual framework for assessing the impact of medical donations (Fig. 1). Data collected as part of a medical donation program can be classified based on whether they describe the resources used, the outcome observed at the program level, or the impact observed at the population level. Examples of input indicators at the program level include data on human and financial resources, quantity of products distributed, and policies identified to initiate the program. Examples of process indicators include whether the donations were delivered on time, whether other planned related activities were carried out as intended, and how well planned activities were carried out. Examples for output measures at the program level include utilization, accessibility, and quality. Outcome, or impact, is defined as changes in population health that can be attributed to the program. Examples of impact measures include mortality, morbidity, and disability-adjusted life-years. Unlike other indicators, evaluation of impact is typically based on models of cause and effect and requires a counterfactual to control for factors other than the intervention that might account for the observed change in population health.

## Results

Twenty-five out of 36 PQMD organizations that were members at the time of the study completed the survey, for a response rate of 69 %. Among the 25 organizations that completed the survey, 12 were corporations and 13 were non-governmental organizations. Twenty-one out of 25 organizations reported having been involved in providing medical donations for over 20 years. Similarly, 21 organizations reported having a person dedicated to managing medical donations. Eighty-eight percent of organizations reported having an internal policy on medical donations. Thirty-six percent of organizations reported having a publicly available external policy on medical donations.

Respondent organizations provided information on a total of 34 donation programs. Characteristics of these donation programs are summarized in Table 1. Thirty-three out of 34 donation programs were ongoing as of the date of the survey. Forty-four percent of donation programs have been operating for over a decade, while 29 % were initiated within the past five years. These programs were reportedly described by respondents because they meet a major unmet need, have the most units donated, are of strategic importance to the organization, are the longest or are most costly. The reported goals of these donation programs included donating medicines, equipment, and funding; providing direct care to patients; addressing rare diseases; educating healthcare professionals, volunteers, technicians, and patients; and managing supply chains. Sub-Saharan Africa and Latin America were the two regions most frequently targeted by the donation programs. Thirteen out of 34 donation programs targeted less than or equal to five different countries. Seven targeted over 50 countries. Recipient country coordination of donations was usually through local hospitals and medical professionals, host-nation Ministry of Health, regional or country office of the organization, and host-nation NGOs.

**Table 1:** Donation program characteristics by whether an impact evaluation was reported

	Total (*n* = 34)
Year program was initiated	
2005 and before	15 (44 %)
2006–2010	7 (21 %)
2011–2015	10 (29 %)
Don’t know	2 (7 %)
Reasons considered a major donation program	
Addresses a major unmet need	26 (76 %)
Has the most units donated	18 (53 %)
Of strategic importance to the organization	18 (53 %)
One of the longest	14 (41 %)
One of the most costly	8 (24 %)
Has the most employees work on it	7 (21 %)
Other^a^	5 (15 %)
Types of events targeted by donation	
Ongoing unmet needs in low-resource settings	23 (68 %)
Strengthening or rebuilding healthcare infrastructures	14 (41 %)
Natural disaster	9 (26 %)
Epidemics	8 (24 %)
Complex emergencies, conflict, war	6 (18 %)
Displaced populations, refugee support	6 (18 %)
Famine, food insecurity	2 (7 %)
Other^b^	7 (21 %)
Types of products donated	
Medical devices, medical equipment	23 (68 %)
Anti-infectives	14 (41 %)
Medical supplies	13 (38 %)
Analgesics	11 (32 %)
Nutritional	10 (29 %)
Respiratory	9 (26 %)
Skin	9 (26 %)
Gastrointestinal	7 (21 %)
Vaccines	7 (21 %)
Oncology medications	6 (18 %)
Oral health	5 (15 %)
Diabetes medications	3 (9 %)
Vector control	2 (6 %)
Other^c^	4 (12 %)
Estimated fair market value (FMV) of donations^d^	
≥$50,000,000	9 (26 %)
$25,000,000–$49,999,999	6 (18 %)
$5,000,000–$24,999,999	5 (15 %)
$1,000,000–$4,999,999	7 (21 %)
<$1,000,000	3 (9 %)
Don’t know	4 (12 %)

^a^Other reasons that were mentioned in the responses included combining equipment and clinical training; maintaining customer relations; strengthening healthcare system; historical involvement with the disease; and involving a reliable, capacity building partner

^b^Other types of events included continuing education; support to frontline health workers; rare diseases; and breast cancer

^c^Other types of donated products included anesthetics; medicines for cardiovascular diseases; medicines for mental illnesses; ophthalmic medicines; and enzyme replacement therapies for rare diseases

^d^Some organizations used internal formulas or list prices to calculate the FMVs for donated products

Donations consisted of a wide range of medical products and services (Table 1). Medical devices, anti-infectives, analgesics, and medical supplies were among the most frequently donated products. The estimated fair market value (FMV) for the donated products for these programs ranged from under one million to over 50 million US dollars (USD). Nine programs donated products that were reportedly valued at more than 50 million USD. The most common estimation method for the FMV was the wholesale acquisition cost (WAC), with 16 programs reporting having used this estimation method. Some organizations also reported using internal formulas or list prices to calculate the FMVs for donated products.

Seventeen out of 34 (50 %) donation programs reported conducting training as a part of their donation program (Table 2). Trainings were provided in the topic areas of disease diagnosis and treatment, nursing skills, maternal and neonatal care, pharmaceutical products usage, mass drug administration, waste management, healthcare facility management, supply chain management, health worker safety, application for drug donations, and program monitoring and evaluation. The format of trainings usually consisted of classroom training, mentorships, or virtual training. External groups involved in providing the training included donor partners, local and international universities, US-based medical research groups, host-nation ministries of health, international organizations, and external NGOs.

**Table 2:** Training, monitoring, and impact evaluations

	Frequency (*n* = 34)
Training conducted as part of the donation program	
Yes	17 (50 %)
Program monitoring conducted	
Yes	26 (76 %)
Phase when monitoring plan was developed	
Inception of the program	13 (38 %)
During the program	11 (32 %)
After products were donated or distributed	12 (35 %)
Impact evaluations conducted	
Yes	10 (29 %)
Phase when impact evaluation was developed	
Inception of the program	6 (18 %)
During the program	6 (18 %)
After products were donated or distributed	4 (12 %)
Cost of impact evaluation	
≤$50,000	7 (21 %)
$50,001–$100,000	0
$100,001–$250,000	2 (6 %)
$250,001–$500,000	0
>$500,000	1 (3 %)
Reasons for not conducting impact evaluations	
Lack of technical staff to conduct impact evaluation	9 (26 %)
Lack of funding	6 (18 %)
Lack of donor interest	2 (6 %)
Lack of CO or NGO interest	3 (9 %)

Of the 34 donation programs, 10 (29 %) were reported to have been evaluated for their impact Each of the organizations that reported conducting an impact evaluation worked in the area of medication donation for more than two decades and reported key staff were devoted to managing medical donations. The longer an organization was engaged in medical donation programs, the greater likelihood that a rigorous evaluation was conducted, as was having staff dedicated to the medical donations program. Two key barriers to conducting impact evaluations for medical donation programs reported by the respondents were lack of technical staff and lack of funding. Impact evaluations that met stakeholders’ needs were often reported to be “very costly”, and some organizations indicated they could not afford such impact evaluations. Seven out of the 10 impact evaluations in this survey were reported to cost less than or equal to 50,000 USD. Impact evaluations were conducted by internal evaluation departments, local and international universities, recipient health facilities, and external NGOs. Similar to monitoring plans, impact evaluations were developed at various phases of the program.

Metrics chosen for impact evaluations depended on the nature of the medical donations. Some examples of reported metrics were quantity of donations; number of patients receiving and benefiting from the treatment; improvement in knowledge and skills; usefulness of training; deficits in budgets of the ministry of health; and participating health facilities.

Findings from impact evaluations were reported to have been disseminated to key stakeholders and the general public through periodic reports, end-user reports, periodic meetings, presentations at forums and conferences, websites and other social media, and scientific publications. Findings were reportedly used to improve the donation program, set the stage for establishing future partnerships, demonstrate continual improvement of internal process and commitment to patients and healthcare, and improve donor-recipient relationships and encourage increased quantity and improved quality medical donations.

## Discussion

Findings from this survey demonstrate that responding organizations provide a broad range of medical donations, targeting a wide range of public health issues and events. Nearly 80 % of the donation programs in this study reported having conducted some level of monitoring and evaluation. However, the types of metrics used in reported evaluations varied greatly. Units of donation and number of patients receiving the donation were often reported to be tracked in an ongoing fashion since they are generally more readily available. However, a program’s impact at the population level was infrequently evaluated. When a program was evaluated, metrics chosen depended on the nature of the medical donations, and some epidemiological and economic outcomes were reported to have been used by a few member organizations.

Most of the impact evaluations reported in this study were relatively small in scale, costing under $50,000. Some organizations indicated that with limited resources, they could only afford small-scale evaluations of the donation programs, although these evaluations may not fully meet stakeholders’ needs. Lack of technical staff and lack of funding were cited as key barriers to conducting a rigorous impact evaluation, despite of a considerable amount of interest in it among PQMD member organizations.

While the survey provides a baseline assessment of past and current evaluations, there were some limitations to this survey. The donation programs described in this study are not representative of the full range of donation programs among the organizations surveyed, nor do they represent the totality of medical donation programs in general, and thus should not be generalized as such. The survey was limited to asking about major donation programs, and the judgment of whether a donation program can be considered a major one was left to the respondents. While the purpose of this survey was to provide insights into the breadth and depth of medical donation programs and evaluations by PQMD members, the survey was not tailored to a specific type of donation or organization. As a result, some respondents may have found some questions not applicable to their organizations or their donation programs. Finally, while a 69 % response rate is generally recognized as acceptable, a higher response rate would have provided more confidence in the generalizability of our results and reduced the likelihood of non-response bias.

## Conclusions

An evaluation should not be an end in itself but rather a means to an end. Factors in deciding when to do an impact evaluation should include the need to demonstrate the impact to key stakeholders, the availability of resources to collect and analyze necessary data, and the stage of the program. All types of programs can benefit from sound monitoring and evaluation, and this includes developing a well thought out analysis plan. Findings from well-conducted impact evaluations can help with making decisions about programs, practices, and policies, and would benefit both donors and recipients of medical donation programs.
